# Electronic Vapor Products: Alarming Trends in United States Adolescents

**DOI:** 10.31486/toj.24.0004

**Published:** 2024

**Authors:** Charles H. Hennekens, Adedamola Adele, Maria C. Mejia, Robert S. Levine, Panagiota Kitsantas

**Affiliations:** ^1^ Department of Medicine, Charles E. Schmidt College of Medicine, Florida Atlantic University, Boca Raton, FL; ^2^ Department of Biomedical Science, Charles E. Schmidt College of Medicine, Florida Atlantic University, Boca Raton, FL; ^3^ Department of Population Health and Social Medicine, Charles E. Schmidt College of Medicine, Florida Atlantic University, Boca Raton, FL; ^4^ Department of Family and Community Medicine, Baylor College of Medicine, Houston, TX

**Keywords:** *Adolescent*, *e-cigarette*, *electronic nicotine delivery systems*, *vaping*

## Abstract

**Background:** The use of electronic vapor products (EVPs) increases the risks of nicotine addiction, drug-seeking behavior, mood disorders, and avoidable premature morbidities and mortality. We explored temporal trends in EVP use among US adolescents.

**Methods:** We used data from the Youth Risk Behavior Survey for school grades 9 through 12 from 2015 (earliest available data) to 2021 (the most recently available data) from the US Centers for Disease Control and Prevention (n=57,006).

**Results:** Daily use of EVPs increased from 2.0% in 2015 to 7.2% in 2019, a greater than 3.5-fold increase. Although the percentage decreased to 5.0% in 2021, it was still a >2.5-fold increase since 2015. In 2015, the percentage of EVP use was significantly higher in boys (2.8%) than girls (1.1%). By 2021, the percentage of EVP use was higher in girls (5.6%) than boys (4.5%), a 1.24-fold increase. In addition, the percentage of EVP use in 2021 was higher in White youth (6.5%) vs Black (3.1%), Asian (1.2%), and Hispanic/Latino (3.4%) youth compared to 2015, but White and Black adolescents had the highest increases of approximately 3.0-fold between 2015 and 2021. Adolescents in grade 12 had the highest percentages of EVP use at all periods.

**Conclusion:** These data show alarming statistically significant and clinically important increases in EVP use in US adolescents in school grades 9 through 12. The magnitude of the increases may have been blunted by coronavirus disease 2019, a hypothesis that requires direct testing in analytic studies. These trends create clinical and public health challenges that require targeted interventions such as mass media campaigns and peer interventions to combat the influences of social norms that promote the adoption of risky health behaviors during adolescence.

## INTRODUCTION

Since their introduction in 2007, electronic vapor products (EVPs), also known as e-cigarettes or vaping devices, have impacted nicotine consumption among adults and adolescents in the United States. In 2022, 6% of US adults reported current EVP use,^1^ and the widespread use of EVPs by adults has raised concerns about use among adolescents. Much of the allure of EVPs is attributable to their marketed image as a safer alternative to traditional cigarette smoking and the variety of EVP flavors that appeal to a broad audience.^2,3^ The use of EVPs markedly increases the acute risks of nicotine addiction, drug-seeking behavior, mood disorders, and long-term risks of avoidable premature morbidities and mortality.^2,4^ In addition, compared to nonusers, adolescents and young adults who use EVPs are more likely to switch to cigarette smoking which, despite remarkable declines in the United States, remains the leading avoidable cause of premature death in the United States and worldwide.^5^

Our previous investigation highlighted a reassuring decline in cigarette smoking among US adolescents.^6^ For this original research study, we used Youth Risk Behavior Survey (YRBS) data for school grades 9 through 12 to explore overall temporal trends in EVP use among US adolescents from 2015 to 2021 (the most recently available data), as well as EVP use by sex (boy/girl), race (Black, Asian, Hispanic/Latino, and White), and grade (9th, 10th, 11th, and 12th).^7^

## METHODS

The YRBS is administered to large samples of US adolescents enrolled in public, parochial, and private schools across the United States and monitors health behaviors that contribute to the leading avoidable causes of premature morbidity and mortality.^7^ Detailed descriptions of the survey have been reported.^8^ The Centers for Disease Control and Prevention conducts the YRBS biennially among students in school grades 9 through 12.

In 2021, the student response rate to the YRBS was 79.1%.^8^ Our study sample consisted of 57,006 adolescents and included data from the years 2015 (n=15,318), 2017 (n=12,844), 2019 (n=12,767), and 2021 (n=16,077).

For the survey, EVP use was defined as the use of e-cigarettes, vapes, vape pens, e-cigars, e-hookahs, hookah pens, and modifiable devices to customize the substance (mods), such as JUUL, SMOK, Suorin, Vuse, and blu. Daily EVP use was defined as use on all 30 days before the survey.

We used differences in percentages as measures of effects. Statistical significance was tested using the 95% CI and the nonparametric chi-squared test that considers a finding significant using a 2-sided *P* value at a level of <0.05. The institutional review board of the Baylor College of Medicine considered this research to be exempt.

## RESULTS

### Overall Trends in Daily EVP Use

Daily use of EVPs rose from 2.0% in 2015 to 7.2% in 2019, a statistically significant (*P* <0.001) and >3.5-fold increase (Figure). The percentage decreased to 5.0% in 2021, which when compared to 2015, still represents a significant >2.5-fold increase in daily EVP use (*P* <0.001).

**Figure.**
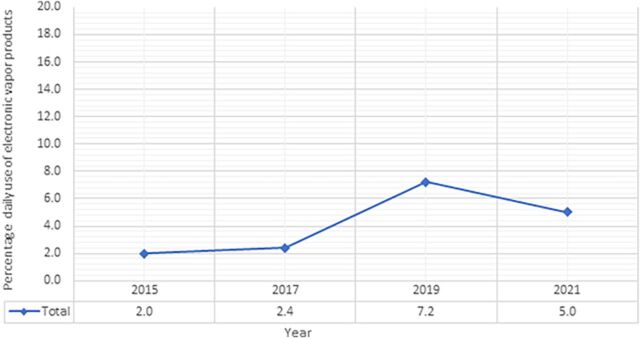
Trends in daily use of **electronic vapor products (EVPs)** in US adolescents. Daily use of EVPs rose significantly (*P* <0.001) from 2.0% in 2015 to 7.2% in 2019. The percentage decreased to 5.0% in 2021, which when compared to 2015, still represents a significant >2.5-fold increase (*P* <0.001).

### Daily EVP Use by Sex

Trends in daily EVP use were significantly higher (*P* <0.001) in boys compared to girls in 2015 and 2019 but were slightly lower by 2021 (Table). For boys, daily EVP use increased significantly (*P* <0.001) from 2.8% in 2015 to 7.9% in 2019, a 2.8-fold increase. The percentage of daily EVP use among boys decreased to 4.5% in 2021, which is still a >1.6-fold increase since 2015. In girls, daily EVP use also increased significantly (*P* <0.001) from 1.1% in 2015 to 6.4% in 2019, a 5.8-fold increase. The percentage decreased in 2021 to 5.6%, which is still a >5.0-fold increase in daily EVP use among girls compared to 2015.

**Table.** Trends in Daily Use of Electronic Vapor Products by Youth Risk Behavior Survey Year^7^

	Use of Electronic Vapor Products, % (95% CI) Survey Year				
Variable	2015	2017	2019	2021	*P* Value
Sex					
Boys	2.8 (2.1-3.5)	3.8 (3.1-4.5)	7.9 (6.7-9.4)	4.5 (3.9-5.2)	<0.001
Girls	1.1 (0.8-1.6)	1.1 (0.7-1.6)	6.4 (5.4-7.6)	5.6 (4.6-6.8)	<0.001
Race/Ethnicity					
Black	1.1 (0.7-1.9)	1.0 (0.5-19)	3.4 (2.0-5.9)	3.1 (2.0-4.7)	0.020
Asian	1.2 (0.5-2.7)	0.6 (0.2-2.0)	3.0 (1.4-6.2)	1.2 (0.5-2.8)	0.150
Hispanic/Latino	2.6 (1.9-3.4)	1.7 (1.2-2.4)	5.2 (4.1-6.6)	3.4 (2.9-4.1)	<0.001
White	1.9 (1.4-2.5)	3.1 (2.4-3.9)	9.3 (8.0-10.9)	6.5 (5.6-7.6)	<0.001
Grade					
9th	1.9 (1.4-2.5)	1.2 (0.8-1.9)	3.6 (2.6-4.8)	2.6 (2.1-3.2)	0.010
10th	1.7 (1.2-2.4)	1.7 (1.2-2.3)	5.4 (3.9-7.3)	3.5 (2.7-4.5)	<0.001
11th	1.8 (1.3-2.5)	2.7 (2.0-3.6)	8.3 (6.7-10.2)	6.0 (4.9-7.3)	<0.001
12th	2.2 (1.4-3.5)	4.0 (2.9-5.4)	12.0 (10.1-14.2)	8.4 (6.9-10.3)	<0.001
Overall	2.0 (1.6-2.5)	2.4 (2.0-3.0)	7.2 (6.2-8.3)	5.0 (4.4-5.7)	<0.001

Note: *P* values were calculated using the Youth Risk Behavior Surveillance System Analysis Tool,^7^ comparing percentages to the 2015 baseline for each variable category.

### Daily EVP Use by Race/Ethnicity

The trends in daily EVP use were similar by race/ethnicity but varied in magnitude between the categories (Table). In 2021, the percentage of daily EVP use was significantly higher among White youths (6.5%) compared to Black (3.1%), Asian (1.2%), and Hispanic/Latino (3.4%) youths (*P* <0.001), but White and Black adolescents had the steepest rises in daily EVP use from 2015 to 2021 of approximately 3-fold. In Black youths, daily EVP use increased significantly from 1.1% in 2015 to 3.4% in 2019, a 3-fold increase, and fell to 3.1% in 2021, which was still a significant 2.8-fold increase since 2015 (*P*=0.020). In Asian youths, daily EVP use increased from 1.2% in 2015 to 3.0% in 2019 (not statistically significant, *P*=0.150), a 2.5-fold increase, and then decreased to the baseline percent of 1.2% in 2021. In Hispanic/Latino adolescents, daily EVP use increased significantly from 2.6% in 2015 to 5.2% in 2019 (*P* <0.001), a 2-fold increase, and dropped to 3.4% in 2021, which was still a 1.3-fold increase compared to 2015. In White adolescents, daily EVP use increased significantly (*P* <0.001) from 1.9% in 2015 to 9.3% in 2019, a 4.9-fold increase, and then declined to 6.5% in 2021 which was still a 3.4-fold increase compared to 2015.

### Daily EVP Use by School Grade

The trends in daily EVP use were similar by school grade (Table). In all 4 survey years, daily EVP use was highest in the 12th grade where most students are aged 17 to 18 years. In 9th graders, daily EVP use increased significantly from 1.9% in 2015 to 3.6% in 2019, a 1.9-fold increase (*P*=0.010), followed by a decrease to 2.6% in 2021, which was still an almost 1.4-fold increase compared to 2015. In 10th graders, daily EVP use increased significantly from 1.7% in 2015 to 5.4% in 2019, an almost 3.2-fold increase (*P* <0.001), with a decrease to 3.5% in 2021 that was still a >2-fold increase since 2015.

In 11th graders, daily EVP use increased significantly from 1.8% in 2015 to 8.3% in 2019, an almost 4.6-fold increase (*P* <0.001), followed by a decline to 6.0% in 2021, which was still a >3.3-fold increase. In 12th graders, daily EVP use also increased significantly from 2.2% in 2015 to 12.0% in 2019, a 5.5-fold increase (*P* <0.001), and then dropped to 8.4% in 2021, which was still a 3.8-fold increase compared to 2015.

## DISCUSSION

These findings show alarming statistically significant and clinically important increases in daily EVP use among US adolescents in the 9th through 12th grades. Daily EVP use decreased from 2019 to 2021, but virtually all percentages remained significantly increased compared to the 2015 baseline. It is tempting to speculate about the role of coronavirus disease 2019 (COVID-19) in explaining these findings. For example, COVID-19 resulted in social distancing, crowd avoidance, and even lockdowns, all of which reduced peer interactions.^9^ Decreased peer interactions and increased parental supervision with remote schooling and work may have led to limited EVP use in this population. Economic constraints and heightened health concerns may have also played a role in decreasing EVP use. These hypotheses, however, need to be tested in analytic studies designed a priori to do so.^10^

Public awareness of the potential dangers associated with the use of EVPs is increasing. A 2023 report concerned a medical condition called e-cigarette or vaping use-associated lung injury (EVALI) that may be caused by the vitamin E acetate used in tetrahydrocannabinol-containing e-cigarettes.^11^ The emergence of EVALI emphasizes the importance of regulatory oversight and consumer awareness. In addition, the legal challenges faced by companies such as JUUL for allegedly marketing to minors (the legal age to purchase e-cigarettes is 21 years), and the enactment of state-level bans on the sale of flavored EVPs have received widespread publicity.

Tobacco use among high school students in the United States has evolved with the advent of EVPs, leading to a complex public health scenario. While data indicate a substantial decline in traditional cigarette smoking among adolescents,^7^ the introduction and spread of EVPs have presented new challenges. The increase in vaping, perceived by some as a safer alternative to smoking, may have contributed to the decline in combustible tobacco product use but has raised concerns about new health risks, including nicotine addiction. Almost 100% of e-cigarettes sold in the US contain nicotine,^12^ and the use of these products by adolescents may lead to future abuse of and addiction to additional substances.^13^ EVPs contain a myriad of substances beyond nicotine, including propylene glycol, glycerin, flavorings, and potentially harmful chemicals such as formaldehyde and metals, which could pose significant health risks.^12^ Moreover, nicotine has a variety of acute adverse effects, and long-term use increases the risks of respiratory diseases, cancer, and cardiovascular diseases.^14^ This complex risk profile underscores the need for stringent research and regulations to address the health impacts of EVP use, especially among vulnerable young populations.

The risks associated with EVP use by adolescents underscore the importance of targeted interventions. In a cross-sectional analysis of data from the Population Assessment on Tobacco and Health study, 44.5% of adolescent EVP users reported that they were seriously interested in quitting, and almost one-quarter had tried to quit during the prior year.^15^ These findings suggest considerable interest among adolescent EVP users to quit, indicating a receptive audience for cessation initiatives or intervention programs targeted at adolescents. Such initiatives/programs could leverage this interest to provide the support and resources needed to help adolescents successfully quit vaping, thereby mitigating the long-term health risks associated with continued use of EVPs.

A strength of the present study is the large sample size. In addition, the YRBS is considered the largest public health surveillance system in the United States, and the survey was conducted during the US epidemic of COVID-19.^8^ YRBS data are weighted to adjust for nonresponse and clustering so that the sample of students is representative of the population. However, our study design is descriptive and therefore useful to formulate, but not to test, hypotheses.^10^

While further research is warranted, the findings from this study highlight the need for targeted prevention and intervention efforts. A 2024 review that assessed vaping prevention interventions for youth found high perceived parental monitoring effective at the individual level.^16^ School-based programs showed inconsistent outcomes, although some social-emotional and peer leadership approaches showed promise in preventing EVP adoption among adolescents.^16^ Reliable evidence of the impact of community interventions is lacking. These descriptive study findings illustrate the need for analytic studies to confirm or refute the effectiveness of these interventions in preventing youth vaping.

Continued surveillance of EVP use in adolescent populations may yield important information for health care practice and policy, including guidance for prevention and control strategies. The interest in quitting expressed by adolescent EVP users^15^ points to a critical window for clinical interventions, including routine screening for vaping and nicotine dependence during adolescent health assessments, counseling, and tailored cessation programs. Given the adverse health effects associated with long-term nicotine use, such as increased risks of respiratory diseases, cancer, and cardiovascular disease, early and proactive clinical engagement is essential. Health care providers play a pivotal role in mitigating the impact of EVP use through education, support for cessation efforts, and advocacy for evidence-based public health policies. The literature indicates the importance of a multifaceted clinical approach to prevent initiation and promote cessation of EVP use among adolescents, thereby contributing to the broader goal of safeguarding adolescent health and well-being.

## CONCLUSION

The findings from this study of daily EVP use among US adolescents from 2015 to 2021 pose important clinical challenges and opportunities in addressing nicotine addiction in this vulnerable population. The sustained high levels of EVP use, despite the observed decrease during the US COVID-19 epidemic, suggest an urgent need for health care providers to integrate routine screening for vaping and nicotine dependence into adolescent health assessments.

## Notes

*This article meets the Accreditation Council for Graduate Medical Education and the American Board of Medical Specialties Maintenance of Certification competencies for Patient Care and Medical Knowledge.*
